# Bias correction methods for test-negative designs in the presence of misclassification

**DOI:** 10.1017/S0950268820002058

**Published:** 2020-09-08

**Authors:** A. Endo, S. Funk, A. J. Kucharski

**Affiliations:** 1 Department of Infectious Disease Epidemiology, London School of Hygiene & Tropical Medicine, Keppel St., London WC1E 7HT, UK; 2 Centre for the Mathematical Modelling of Infectious Diseases, London School of Hygiene & Tropical Medicine, Keppel St., London WC1E 7HT, UK; 3 The Alan Turing Institue, Euston Rd., London NW1 2DB, UK

**Keywords:** Misclassification bias, sensitivity, specificity, test-negative design, vaccine effectiveness

## Abstract

The test-negative design (TND) has become a standard approach for vaccine effectiveness (VE) studies. However, previous studies suggested that it may be more vulnerable than other designs to misclassification of disease outcome caused by imperfect diagnostic tests. This could be a particular limitation in VE studies where simple tests (e.g. rapid influenza diagnostic tests) are used for logistical convenience. To address this issue, we derived a mathematical representation of the TND with imperfect tests, then developed a bias correction framework for possible misclassification. TND studies usually include multiple covariates other than vaccine history to adjust for potential confounders; our methods can also address multivariate analyses and be easily coupled with existing estimation tools. We validated the performance of these methods using simulations of common scenarios for vaccine efficacy and were able to obtain unbiased estimates in a variety of parameter settings.

## Introduction

Vaccine effectiveness (VE) is typically estimated as the vaccine-induced risk reduction of the target disease (TD) and has been traditionally studied using cohort or case–control designs. However, the test-negative design (TND) is becoming a popular alternative design for VE studies [1, 2]. This is a modified version of the case–control study with an alternative definition of the control group; traditional case–control studies usually define controls as non-disease individuals in the study population, while TND studies use individuals with similar symptoms to the TD but presenting negative test results (i.e. patients of non-target diseases; ND). The TND can therefore minimise ascertainment bias by including only medically-attended patients in both case and control groups. Many TND studies have focused on influenza vaccination, but recent studies have also considered other diseases including pneumococcal disease [3, 4] and rotavirus disease [5–7].

Despite its increasing popularity, a TND can be more vulnerable than other study designs to misclassification of disease outcome. Multiple studies have shown that VE is underestimated when the diagnostic tests used in the study are imperfect (i.e. have a sensitivity and/or a specificity less than 100%) [8–10]. This can be a particular issue when simple tests (e.g. rapid diagnostic tests) are used for logistical convenience, as simple tests tend to have lower diagnostic performance than more advanced tests (e.g. polymerase chain reaction; PCR). Previous studies evaluated the expected degree of bias and concluded that specificity had a more important effect on bias than sensitivity [8–11]. These findings appear to support the use of rapid tests, despite limited sensitivity, because the specificity of these tests is typically high [2]. However, theoretical studies to date have been based on a limited range of assumptions about efficacy and pathogen epidemiology; it is therefore unclear whether such conclusions hold for all plausible combinations of scenarios.

If a study is expected to generate a non-negligible bias in estimation, such bias needs to be assessed and – if possible – corrected before the estimate is reported. Greenland [12] proposed a bias correction method for cohort studies where the sensitivity and specificity of the test are known (or at least assumed). However, it has been pointed out that bias correction in case–control studies is in general difficult because of differential recruitment, whereby the probability of recruiting (test-positive) cases and (negative) controls may be different [12, 13]. Although TND studies are often considered to be special cases of case–control studies, they are free from the issue of differential recruitment because the recruitment and classification are mutually-independent [14]. This means that, while Greenland's method does not apply to TND as-is, another type of bias correction may still be possible. For example, De Smedt *et al*. have characterised the misclassification bias in VE in the TND in a simulation study [10]. One limitation of their formulation was it relies on the unobserved âœtrueâ disease risk being known, where in reality this is not usually measurable in field studies. As a result, bias correction methods for TND studies that are directly applicable to field data have not yet been proposed. Moreover, previous analysis of misclassification bias has not considered the impact of multivariate analysis, where potential confounders (e.g. age and sex) are also included in the model used to estimate VE.

To address these issues, we develop a bias correction method for the test-negative VE studies that uses only data commonly available in field studies. We also apply these methods to multivariate analyses. As our approach uses the so-called multiple overimputation (MO) framework (generalisation of multiple imputation) [15], it can easily be coupled with a wide range of estimation tools without modifying their inside algorithms. Finally, we evaluate the performance of our methods by simulations of plausible epidemiological scenarios.

## Methods and results

### Characterising bias in TND studies

First, we consider the case where only vaccination history is included as a risk factor of acquiring the TD (i.e. the univariate setting). Following the approach of Haber *et al*. [16], we consider four steps in the case reporting process: vaccination, onset of symptoms, seeking of medical care and diagnosis. For simplicity, let us assume that occurrence of TD and ND are mutually independent, where their prevalences in the unvaccinated population are represented as *r*_1_ and *r*_0_, respectively^1^. Let *n*_*V*_ and *n*_*U*_ be the vaccinated and unvaccinated population size. The target variable in VE studies is *γ*, the relative risk of TD in the vaccinated population relative to the unvaccinated (i.e. VE = 1 − *γ*). Vaccinated and unvaccinated population can have different likelihoods of seeking medical treatment given disease. We denote by *m*_*V*_ and *m*_*U*_ the probability of medical attendance given ND in vaccinated and unvaccinated population, respectively.

As our focus in the present study is the bias in VE estimation caused by imperfect tests, we made two key assumptions following Haber *et al*. [16]. One assumption is that vaccines have no effect on the risk of ND. This enables ND patients to be eligible for a control group and is a key assumption in TND studies. The other assumption is that the probability of medical attendance in vaccinated and unvaccinated population given infection is constant regardless of the disease (TD or ND). The probability of medical attendance given TD may be different from ND (*m*_*V*_ and *m*_*U*_), potentially due to difference in severity; we assume that these probabilities are obtained by multiplying a constant factor *μ* (i.e. *μm*_*V*_ and *μm*_*U*_)^2^. These assumptions may not always hold and TND can be biased in such cases. However, we assume that they do in the following analysis to keep our focus on misclassification bias; namely, the study was assumed to be able to provide an unbiased VE estimate if tests are perfect.

Following the above notations, we can classify the vaccinated and unvaccinated population into multiple categories shown in Table 1. We can characterise different VE study designs (cohort, case–control and test-negative) by the categories in Table 1 from which each design tries to sample: the cohort design samples from populations *n*_*V*_ and *n*_*U*_ and follows them up to see what proportions fall into *x*_*V*_ and *x*_*V*_; the case–control design samples from medically-attended cases (*x*_*V*_ + *x*_*U*_) and non-diseased controls (*n*_*V*_ − *x*_*V*_ + *n*_*U*_ − *x*_*U*_) and calculate the odds ratio to approximate the relative risk (however, the actual studies can mismeasure these variables when misclassificaiton is present).

**Table 1.** Population classified into different categories of interest in VE studies

	Vaccinated		Unvaccinated	
	Notation	Mean	Notation	Mean
All	*n*_*V*_	*n*_*V*_	*n*_*U*_	*n*_*U*_
Infected by TD	−	*γr*_1_*n*_*V*_	−	*r*_1_*n*_*U*_
Medically-attended (true) TD patients	*x*_*V*_	*μm*_*V*_*γr*_1_*n*_*V*_	*x*_*U*_	*μm*_*U*_*r*_1_*n*_*U*_
Test-positive TD patients	*x*_+*V*_	*αμm*_*V*_*γr*_1_*n*_*V*_	*x*_+*U*_	*αμm*_*U*_*r*_1_*n*_*U*_
Test-negative TD patients	*x*_−*V*_	(1 − *α*)*μm*_*V*_*γr*_1_*n*_*V*_	*x*_−*U*_	(1 − *α*)*μm*_*U*_*r*_1_*n*_*U*_
Infected by ND	−	*r*_0_*n*_*V*_	−	*r*_0_*n*_*U*_
Medically-attended (true) ND patients	*y*_*V*_	*m*_*V*_*r*_0_*n*_*V*_	*y*_*U*_	*m*_*U*_*r*_0_*n*_*U*_
Test-positive ND patients	*y*_+*V*_	(1 − *β*)*m*_*V*_*r*_0_*n*_*V*_	*y*_+*U*_	(1 − *β*)*m*_*U*_*r*_0_*n*_*U*_
Test-negative ND patients	*y*_−*V*_	*βm*_*V*_*r*_0_*n*_*V*_	*y*_−*U*_	*βm*_*U*_*r*_0_*n*_*U*_

In TND studies, medically-attended patients (*x*_*V*_ + *x*_*U*_ + *y*_*V*_ + *y*_*U*_) are sampled and classified into four categories based on the test result and vaccine history. Let *q* be the proportion sampled relative to the population. Denoting the observed case counts with misclassification by *X* and *Y*, the process of data collection in TND can be represented by the following matrix expression:
1
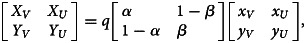
where *α* and *β* are the sensitivity and specificity of the test, respectively. Matrix

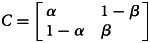
describes the conversion from the true disease state to the observed result. We hereafter refer to *C* as the classification matrix. The determinant *c* = |*C*| = *α* + *β* − 1 is the Youden index of the test and satisfies 0 < *c* ≤ 1 (if *c* < 0, the test is not predictive and the definitions of positive/negative should be swapped). Youden index indicates the level of information retained in the potentially misclassified test results. Youden index of 0 indicates that the information is completely lost and the test is no better than random guesses.

**Fig. 1.**
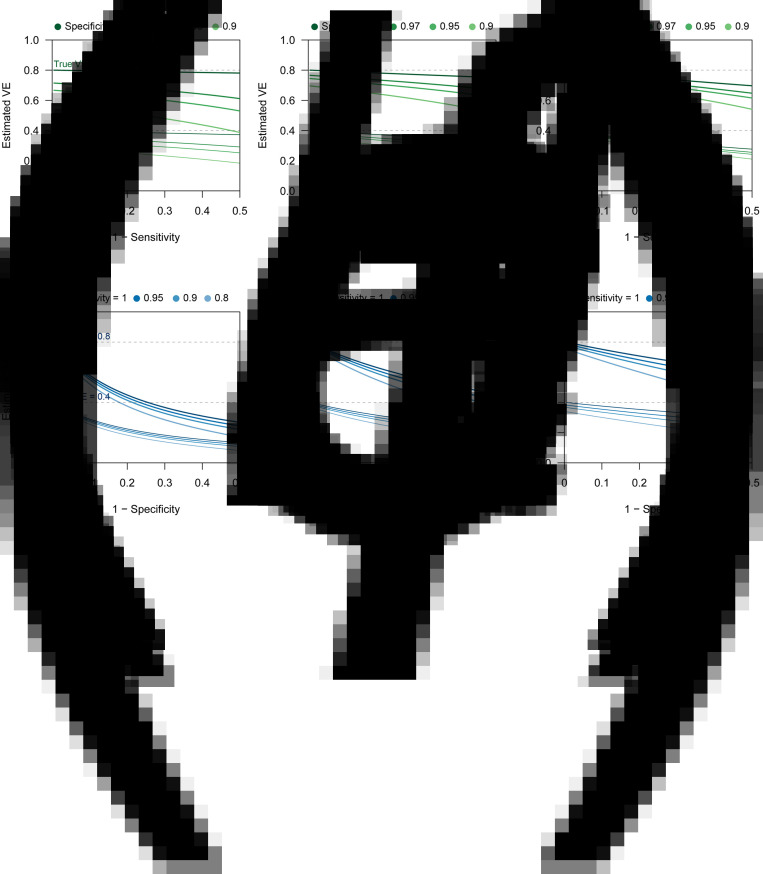
Bias in VE estimates caused by misclassification for different combinations of parameter values. (a)–(c) Estimated VE plotted against sensitivity. (a) True case ratio (the true ratio between TD and ND cases included in the study) = 0.2 (b) 0.4 (c) 0.6. Each two sets of lines respectively correspond to different true VEs (80% and 40%, denoted by the dotted lines). (d)–(f) Estimated VE plotted against specificity. (d) True case ratio = 0.2 (e) 0.4 (f) 0.6.

We define bias in the VE estimate to be the absolute difference between the (raw) estimate, derived from the misclassified observation, and the true value. Let *δ* = ((*r*_1_*μ*)/*r*_0_) be the odds of the (medically-attended) TD in the unvaccinated population. Then the expected bias *B* is given as a function of four independent parameters, *α*, *β*, *γ* and *δ*:
2
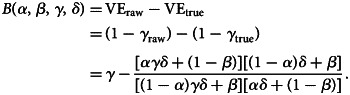
This suggests that the influence of sensitivity/specificity on the degree of bias varies depending on the case ratio *δ*/(1 + *δ*), i.e. the ratio between the incidence of medical attendance for TD and ND in the unvaccinated study population^3^ (Fig. 1). The degree of bias also depends on *γ* but is independent of *m*_*V*_ and *m*_*U*_. The degree of bias is largely determined by the test specificity when the case ratio is small, but the influence of sensitivity and specificity is almost equivalent to a case ratio of 0.6. It is notable that high specificity does not always assure that the bias is negligible. This may be true if specificity is strictly 100% and the case ratio is low to moderate, but a slight decline to 97% can cause a bias up to 10–15 percentage points. The effect of sensitivity is also non-negligible when the case ratio is high.

**Fig. 2.**
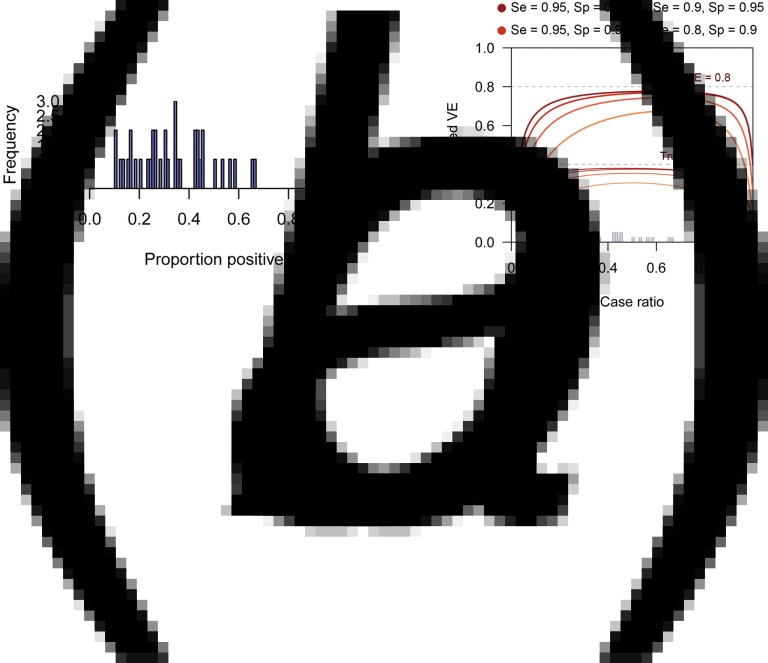
Biased VE estimates with varying case ratio and the observed proportion of positive patients. (a) The proportion of test-positive patients in TND studies from systematic reviews. The proportions were retrieved from three systematic reviews [21–23]. (b) Estimated VE plotted against case ratio. Two sets of lines respectively correspond to different true VEs (80% and 40%, denoted by the dotted lines). The histogram in Panel (a) is overlaid on the *x*-axis.

When the expected bias is plotted against the case ratio with various combinations of test performance, we find that VE estimates can be substantially biased for certain case ratios (especially when the ratio is far from 1:1), even with reasonably high sensitivity and specificity (Fig. 2b). In TND studies, researchers have no control over the case ratio because the study design requires that all tested individuals be included in the study. We found that the proportion of TD-positive patients in previous TND studies (retrieved from three systematic reviews [21–23]) varied considerably, ranging from 10% to 70% (Fig. 2a)^4^. Because of this large variation in the case ratio, it would be difficult to predict the degree of bias before data collection. Post-hoc assessment and correction therefore need to be considered. See the Supplementary Document for further analysis of the degree of bias.

### Bias correction in univariate analysis

#### Corrected odds ratio

Although TND has sometimes been referred to as a special case of the case–control design, there is a distinct feature in the sampling procedure of TND. It has been pointed out that the adjustment methods for misclassification bias developed for cohort studies do not apply to case–control studies because the sampling ratio in case–control studies (differential recruitment) varies between case and control groups [12, 13]. However, as we have shown in Section ‘Characterising bias in TND studies’, both cases (TD patients) and controls (ND patients) are sampled at the same ratio (*q*) in TND studies. This suggests that the existing bias correction formulas developed for a hypothetical setting [13] where the whole population is evenly sampled (which is unrealistic in traditional studies) may be applicable to TND studies.

By left-multiplying Equation (1) with the inverted matrix *C*^−1^, we can obtain the corrected odds ratio γ* as
3
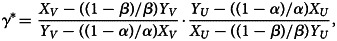
which adjusts for misclassification to give an asymptotically-unbiased estimate of *γ*. This result can also be derived by maximising the likelihood accounting for misclassification in the observed TND data (see the Supplementary Document).

All four components of (3) (two numerators and two denominators) are usually expected to be non-negative with moderate VE (less than 100%) because these components are considered to be proportional to reconstructed true case counts. However, in some (relatively rare) cases, one or more components may become negative due to random fluctuations in observation. Theoretically, negative values are not permitted as true case counts, and thus such negative quantities would need to be truncated to 0. As a result, the corrected odds ratio can be either 0 or infinity. It is unrealistic in clinical settings that vaccines have absolute 100% or −100% effectiveness. Uncertainty around such MLEs should be carefully considered; increasing sample size or redesigning the study might be recommended where possible. Alternatively, the Bayesian framework may be used to yield an interval estimate with the likelihood shown in section ‘Direct likelihood method for the logistic regression model’ adapted for a univariate model.

The confidence interval for VE can be obtained by assuming log-normality of the odds ratio *γ*, i.e.

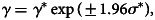
where *σ* is the shape parameter of the log-normal distribution and is empirically given as
4
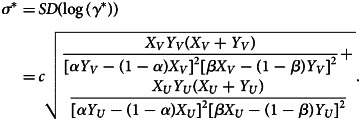


See the Supplementary Document for details of the MLE and confidence intervals.

#### Simulation

To assess the performance of the corrected odds ratio given in Equation (3) and uncertainty around it, we used simulation studies. TND study datasets were drawn from Poisson distributions (see the Supplementary Document for model settings and the likelihood function) as it is a reasonable assumption when medically-attended cases are recruited over the study period. We parameterised the mean incidence in the dataset by the ‘baseline medical attendance’ *λ*_*V*_ = *qm*_*V*_(*r*_1_*μ* + *r*_0_)*n*_*V*_ and *λ*_*U*_ = *qm*_*U*_(*r*_1_*μ* + *r*_0_)*n*_*U*_, so that *λ*_*V*_ and *λ*_*U*_ correspond to the mean number of vaccinated/unvaccinated patients when vaccine has no effect (i.e. *γ* = 1 and VE = 0). The mean total sample size (given as ((1 + *γδ*)/(1 + *δ*))*λ*_*V*_ + *λ*_*U*_) was set to be 3000. Parameter values were chosen according to a range of scenarios shown in Table 2, and the true VE=1−*γ* was compared with the estimates obtained from the simulated data. For each scenario, simulation was repeated 500 times to yield the distribution of estimates. Reproducible codes (including those for simulations in later sections) are reposited on GitHub (https://github.com/akira-endo/TND-biascorrection/).

**Table 2.** Simulation settings

ID	Scenario	True VE (*γ*)	*λ*_*V*_/*λ*_*U*_	Case ratio (*γ*/(1 + *γ*))	Sensitivity (*α*)	Specificity (*β*)
1	Baseline: low VE	0.4	0.5	0.5	0.8	0.95
2	Baseline: high VE	0.8	0.5	0.5	0.8	0.95
3	High quality test: low VE	0.4	0.5	0.5	**0.95**	**0.97**
4	High quality test: high VE	0.8	0.5	0.5	**0.95**	**0.97**
5	Low quality test: low VE	0.4	0.5	0.5	**0.6**	0.9
6	Low quality test: high VE	0.8	0.5	0.5	**0.6**	0.9
7	High TD incidence: low VE	0.4	0.5	**0.7**	0.8	0.95
8	High TD incidence: high VE	0.8	0.5	**0.7**	0.8	0.95
9	Low TD incidence: low VE	0.4	0.5	**0.3**	0.8	0.95
10	Low TD incidence: high VE	0.8	0.5	**0.3**	0.8	0.95
11	High vaccine coverage: low VE	0.4	**0.7**	0.5	0.8	0.95
12	High vaccine coverage: high VE	0.8	**0.7**	0.5	0.8	0.95
13	Low vaccine coverage: low VE	0.4	**0.3**	0.5	0.8	0.95
14	Low vaccine coverage: high VE	0.8	**0.3**	0.5	0.8	0.95

**Fig. 3.**
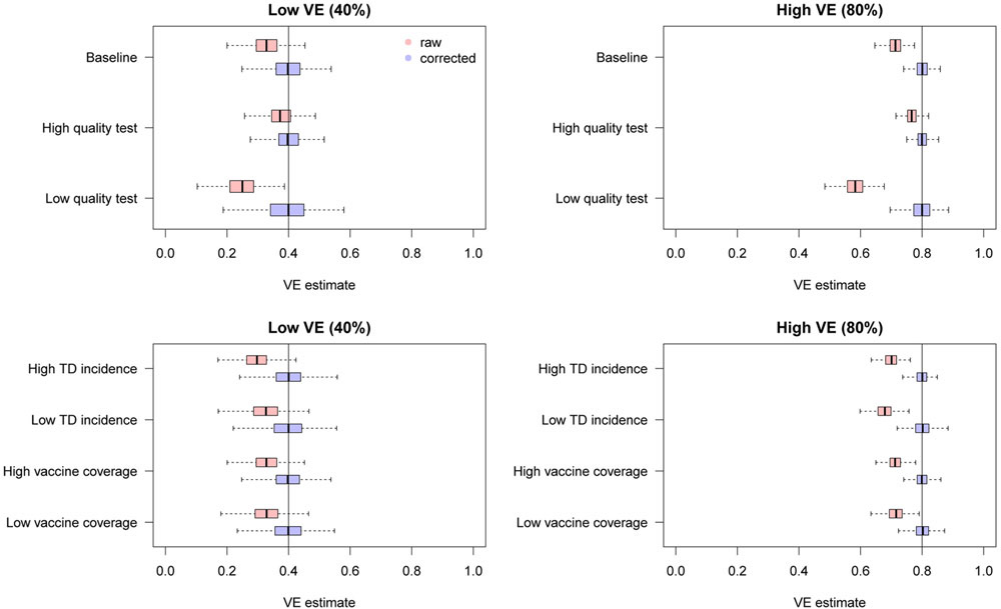
Bias correction for simulated data in the univariate setting. The distributions of bias-corrected VE estimates (boxplots in blue) are compared with those of raw VE estimates without correction (red). Five hundred independent datasets were randomly generated for each set of parameter values, and the corrected and uncorrected VE estimates are compared with the true value (black solid line). See Table 2 for parameter settings in each scenario.

We found that the uncorrected estimates, directly obtained from the raw case counts that were potentially misclassified, exhibited substantial underestimation of VE for most parameter values (Fig. 3). On the other hand, our bias correction method was able to yield unbiased estimates in every setting, whose median almost correspond to the true VE. Although the corrected and uncorrected distributions were similar (with a difference in median ~5%) when VE is relatively low (40%) and the test has sufficiently high sensitivity and specificity (95% and 97%, respectively), they became distinguishable with a higher VE (80%). With lower test performances, the bias in the VE estimates can be up to 10–20%, which may be beyond the level of acceptance in VE studies.

#### Bias correction of VEs reported in previous studies

We have seen that the degree of bias for uncorrected VE estimates depends on parameter values. To explore the possible degree of bias in existing VE studies, we extracted the reported crude VEs (i.e. VEs unadjusted for potential confounders) from two systematic reviews [21, 23]^5^ and applied our bias correction method assuming different levels of test sensitivity and specificity. The case counts for each study summarised in the reviews were considered eligible for the analysis if the total sample size exceeded 200. Varying the assumed sensitivity and specificity, we investigated the possible discrepancy between the reported VE (or crude VE derived from the case counts if unreported in the reviews) and bias-corrected VE. We did not consider correcting adjusted VEs because it requires access to the original datasets.

**Fig. 4.**
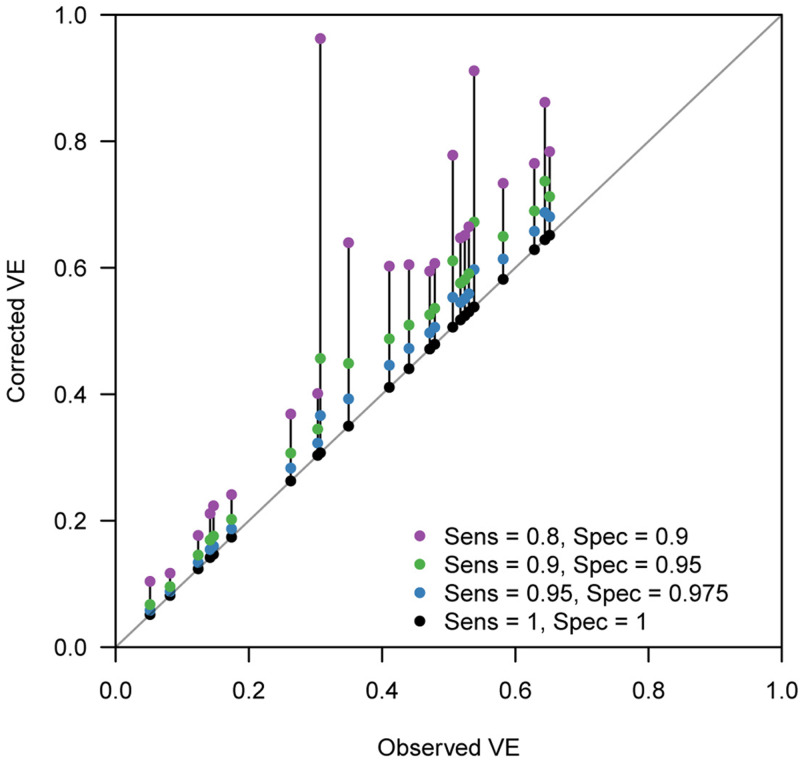
Bias correction method applied to published VE estimates assuming various test sensitivity and specificity. Case count data were extracted from two systematic reviews [21, 23]. Each connected set of dots show how (crude) VE estimates reported in the review varies when imperfect sensitivity and specificity are assumed. Black dots on the grey diagonal line denote the original VEs reported in the reviews. This should correspond to the true value if sensitivity = specificity = 1. Coloured dots show the bias-corrected VE considering potential misclassification.

Figure 4 displays the discrepancy between the reported VE and bias-corrected VE corresponding to a range of assumptions on the test performance. Many of the extracted studies employed PCR for the diagnostic test, which is expected to have a high performance. However, the true performance of PCR cannot be definitively measured as there is currently no other gold-standard test available. Figure 4 suggests that even a slight decline in the test performance can introduce a non-negligible bias in some parameter settings. Our bias correction methods may therefore also be useful in TND studies using PCR, which would enable a sensitivity analysis accounting for potential misdiagnosis by PCR tests. In this light, it is useful that the corrected odds ratio

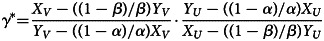
is a monotonic function of both *α* and *β* (given that all the four components are positive). The possible range of VE in a sensitivity analysis is obtained by supplying γ* with the assumed upper/lower limits of sensitivity and specificity.

### Bias correction in multivariate analysis

#### Theoretical framework

TND studies often employ a multivariate regression framework to address potential confounding variables such as age. The most widespread approach is to use generalised linear models (e.g. logistic regression) and include vaccination history as well as other confounding variables as covariates. The estimated linear coefficient for vaccination history can then be converted VE (in the logistic regression model, the linear coefficient for vaccination history corresponds to log (1 − VE)). In this situation, the likelihood function now reflects a regression model and thus the bias-corrected estimate in the univariate analysis (Equation (3)) is no longer applicable. We therefore need to develop a separate multivariate TND study framework to correct for bias in multivariate analysis.

Suppose that covariates *ξ* = (*ξ*^1^, *ξ*^2^, …, *ξ*^*n*^) are included in the model, and that *ξ*^1^ corresponds to vaccination history (1: vaccinated, 0: unvaccinated). These covariates *ξ*_*i*_, as well as outcome variable *Z*_*i*_ (i.e. test results) are available for each individual *i* included in the study. In TND studies, it is often convenient to model the binomial probability for the true outcome *p*_1_(*ξ*_*i*_), i.e. the conditional probability that the true outcome is TD as opposed to ND given an individual has covariates *ξ*_*i*_. Let us use parameter set *θ* to model the binomial probabilities *p*_1_ (and *p*_0_ = 1 − *p*_1_). Using the binomial probability 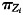 for observed (potentially misclassified) outcome *Z*_*i*_, we can obtain the MLE for *θ* by maximising
5
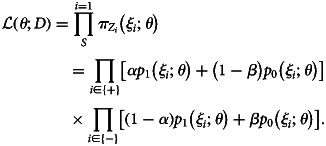


With the estimate θ*, the VE estimate for an individual with covariates *ξ*^2:*n*^ = (*ξ*^2^, *ξ*^3^, …, *ξ*^*n*^) is given as (1−odds ratio):
6
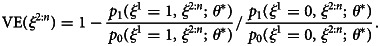


See the Supplementary Document for further details.

#### Direct likelihood method for the logistic regression model

The logistic regression model is well-suited for modelling binomial probabilities *p*_1_ and *p*_0_. The log-odds (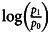) is characterised by a linear predictor as:
7
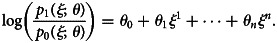
In the logistic regression model where covariate *ξ*_1_ indicates vaccination history, the corresponding coefficient *θ*_1_ gives the VE estimate: VE = 1 − exp (*θ*_1_). Due to the assumed linearity, the estimated VE value is common across individuals regardless of covariates *ξ*^2:*n*^.

We can employ the direct likelihood method by combining Equations (5) and (7). The usual logistic regression optimises *θ* by assuming that the test results follow Bernoulli distributions *Z*_*i*_ ~ Bernoulli(*p*_1_(*ξ*_*i*_;*θ*)) (*Z*_*i*_ = 1 for positive test results and 0 for negative). To correct the misclassification bias, we instead need to use the modified probabilities to construct the likelihood accounting for diagnostic error, i.e.
8
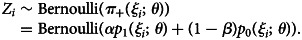
Parameter *θ* is estimated by directly maximising the probability of observing {*Z*_*i*_} based on Equation (8)

Note that as long as the binomial probability is the modelling target, other type of models (e.g. machine learning classifiers) could also be employed under a similar framework.

#### Multiple overimputation combined with existing tools

The direct likelihood method presented in the previous section is the most rigorous MLE approach and would therefore be preferable whenever possible. However, it is often technically-demanding to implement such approaches as it involves re-defining the likelihood; if we wanted to use existing tools for logistic regression (or other models), for example, we would need to modify the internal algorithm of such tools. This is in particular complicated in tools for generalised linear models including logistic regression, whose standard algorithm is the iteratively reweighted least squares method [24], which does not involve the explicit likelihood. To ensure that our correction methods can be employed without losing access to substantial existing software resources, we also propose another method, which employs a MO framework [15] to account for misclassification. Whereas multiple imputation only considers missing values, MO is proposed as a more general concept which includes overwriting mismeasured values in the dataset by imputation. In our multivariate bias correction method, test results in the dataset (which are potentially misclassified) are randomly overimputed.

Let *M* be an existing estimation software tool whose likelihood specification cannot be reprogrammed. Given data *d* = {*z*_*i*_, *ξ*_*i*_}_*i*=1,2,…*S*_, where *z*_*i*_ denotes the true disease state (*z* = 1 for TD and *z* = 0 for ND), *M* would be expected to return at least the following two elements: the point estimate of VE (*ɛ*_*d*_) and the predicted binomial probability 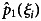 for each individual *i*. From the original observed dataset *D*, *J* copies of imputed datasets 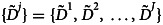 are generated by the following procedure.

1. For *i* = 1, 2, …, *S*, impute disease state 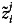 based on the test result *Z*_*i*_. Each 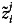 is sampled from a Bernoulli distribution conditional to *Z*_*i*_:
9
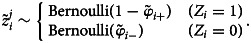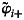 and 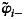 are estimated probabilities that the test result for individual *i* is incorrect (i.e. *z*_*i*_ ≠ *Z*_*i*_) given *Z*_*i*_. The sampling procedure (9) is therefore interpreted as the test result *Z*_*i*_ being ‘flipped’ at a probability 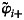 or 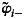. Later we will discuss possible procedures to obtain these probabilities.
3. Apply *M* to 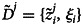 to yield a point estimate of VE (*ɛ*^*j*^).
4. Repeat (1) and (2) for *j* = 1, 2, …, *J* to yield MO estimates {*ɛ*^*j*^}_*j*=1,…*J*_.

Once MO estimates {*ɛ*^*j*^} are obtained, the pooled estimate and confidence intervals of VE are obtained by appropriate summary statistics, e.g. Rubin's rules [25]. As long as the estimated ‘flipping’ probabilities 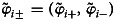 are well chosen, this MO procedure should provide an unbiased estimate of VE with a sufficiently large number of iterations *J*.

As a method to estimate the flipping probability 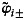, here we propose the parametric bootstrapping described as follows. Given the observed test result *Z*_*i*_, 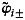 is given as the Bayesian probability
10
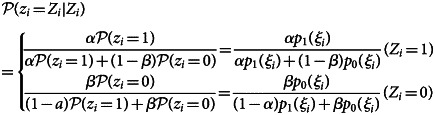


**Fig. 5.**
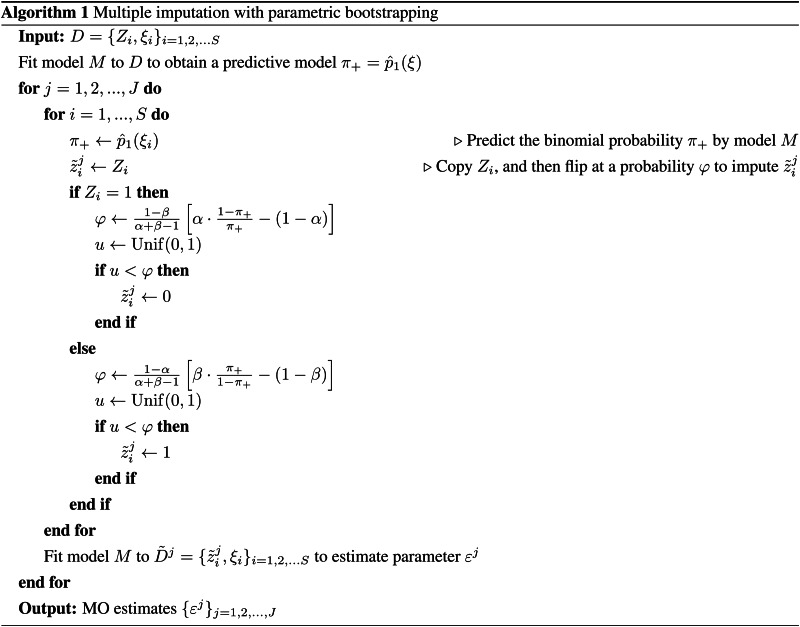
Multiple imputation with parametric bootstrapping.

Although the true binomial probabilities *p*_0_(*ξ*_*i*_), *p*_1_(*ξ*_*i*_) are unknown, their estimators are derived with the inverted classification matrix in the same manner as Equation (3). By substituting

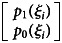
with

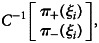
we get
11
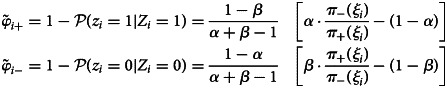
These probabilities can be computed provided the odds of the test results *π*_+_ (*ξ*_*i*_)/*π*_−_(*ξ*_*i*_). We approximate this odds by applying estimation tool *M* to the original data *D*; i.e. the predicted binomial probability 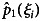 obtained from *D* is used as a proxy of *π*_+_ (*ξ*_*i*_). Generally it is not assured that true and observed probabilities *p*_1_(*ξ*_*i*_) and *π*_+_ (*ξ*_*i*_) have the same mechanistic structure captured by *M*; however, when our concern is limited to the use of model-predicted probabilities to smooth the data *D*, we may expect for *M* to provide a sufficiently good approximation. The above framework can be regarded as a variant of parametric bootstrapping methods as MO datasets are generated from data *D* assuming a parametric model *M*. The whole bias correction procedure is presented in pseudocode (Fig. 5); sample R code is also available on GitHub (https://github.com/akira-endo/TND-biascorrection/).

#### EM algorithm

Another possible approach to addressing misclassification is the use of the EM algorithm, which has been proposed for case–control studies in a previous study (where differential recruitment was not considered) [26]. Because of its methodological similarity, the algorithm can also be applied to the TND. The original EM algorithm presented in [26] would produce, if properly implemented, the result equivalent to the direct likelihood approach. However, the original EM algorithm requires that the model can handle non-integer sample weights (which may not always be assured). Moreover, computing confidence intervals in EM algorithm can be complicated. We therefore recommend parametric bootstrapping as the first choice of bias correction method when the direct likelihood approach is inconvenient.

#### Simulation of bias correction with parametric bootstrapping

To assess the performance of this method, we used the same simulation framework as in the univariate analysis (Table 2). In addition to vaccination history (denoted by *ξ*^1^), we consider one categorical and one continuous covariate. Let us assume that *ξ*_2_ represents the age group (categorical; 1: child, 0: adult) and *ξ*_3_ the pre-infection antibody titre against TD (continuous). Suppose that the population ratio between children and adults is 1:2, and that *ξ*_3_ is scaled so that it is standard normally distributed in the population. For simplicity, we assumed that all the covariates are mutually independent with regard to the distribution and effects (i.e. no association between covariates and no interaction effects). The relative risk of children was set to be 2 and 1.5 for TD and ND, respectively, and a unit increase in the antibody titre was assumed to halve the risk of TD (and not to affect the risk of ND). The mean total sample size *λ* was set to be 3000, and 500 sets of simulation data were generated for each scenario. VE estimates were corrected by the parametric bootstrapping approach (the number of iterations *J* = 100) and were compared with the raw (uncorrected) VE estimates.

**Fig. 6.**
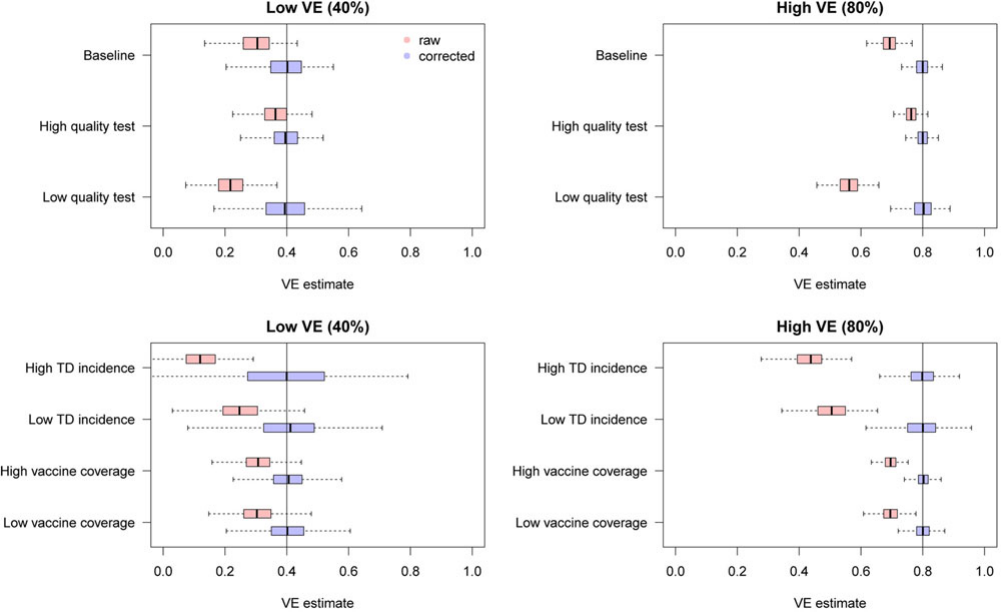
Bias correction for simulated data in the multivariate setting. The distributions of bias-corrected (blue) and uncorrected (red) VE estimates from 500 simulations are compared. Dotted lines denote median and black solid lines denote the true VE. The parametric bootstrapping bias correction method was used for bias correction.

Figure 6 shows the distributions of estimates with and without bias correction in the multivariate setting. Our bias correction (parametric bootstrapping) provided unbiased estimates for all the scenarios considered. Overall, biases in the uncorrected estimates were larger than those in the univariate setting. In some scenarios, the standard error of the bias-corrected estimates was extremely wide. This was primarily because of the uncertainty already introduced before misclassification rather than the failure of bias correction (as can be seen in the Supplementary Fig. S3). Larger sample size is required to yield accurate estimates in those settings, as the information loss due to misclassification will be added on top of the inherent uncertainty in the true data.

#### The number of confounding variables

We investigated how the bias in uncorrected VE estimates can be affected by the number of confounding variables. In addition to the vaccine history *ξ*^1^, we added a set of categorical/continuous confounding variables to the model and assessed the degree of bias caused by misclassification. The characteristics of the variables were inherited from those in section ‘Simulation of bias correction with parametric bootstrapping’: categorical variable ‘age’ and continuous variable ‘pre-infection antibody titre’. That is, individuals were assigned multiple covariates (e.g. ‘categorical variable A’, ‘categorical variable B’, …, ‘continuous variable A’, ‘continuous variable B’, …) whose distribution and effect were identical to ‘age’ (for categorical variables) and ‘antibody titre’ (for continuous variables) in section ‘Simulation of bias correction with parametric bootstrapping’. No interaction between covariates was assumed. The covariate set in section ‘Simulation of bias correction with parametric bootstrapping’ being baseline (the number of covariates: (vaccine, categorical, continuous) = (1, 1, 1)), we employed two more scenarios with a larger number of covariates: (1, 3, 3) and (1, 5, 5).

**Fig. 7.**
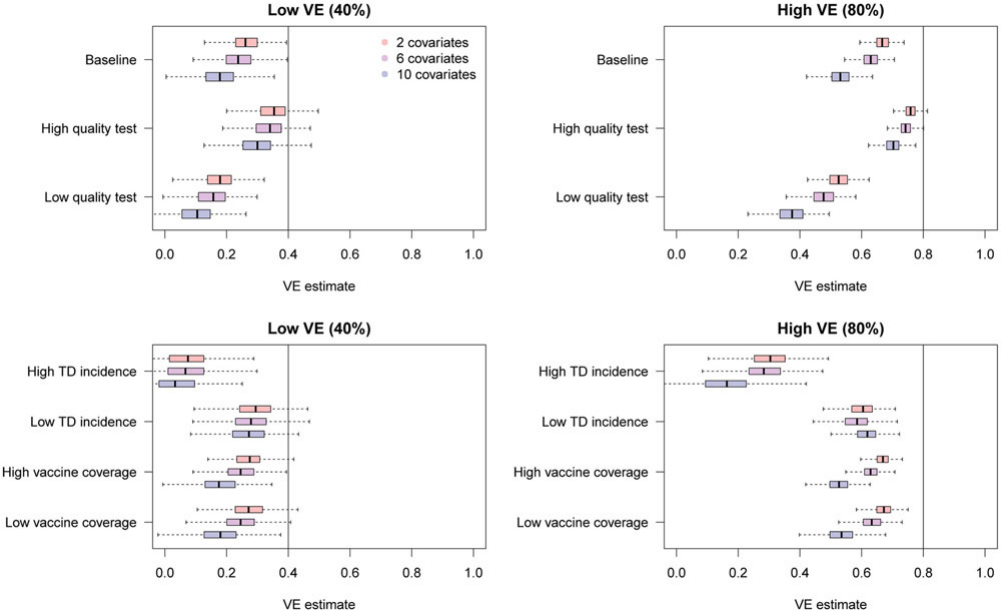
Bias in raw VE estimates from simulated data in the presence of different numbers of confounding variables. The distributions in red, purple and blue correspond to uncorrected VE estimates in the presence of 2, 6 and 10 confounding variables in addition to the vaccination history.

The simulation results are presented in Figure 7. Overall, additional confounding variables led to more severe bias in the uncorrected VE estimates towards underestimation. These results further highlight the importance of bias correction when heterogeneous disease risks are expected; VE estimates adjusted for many confounding variables can exhibit substantial misclassification bias.

## Discussion

Misclassification caused by imperfect diagnostic tests can potentially lead to substantial biases in TND studies. By considering the processes involved in VE estimation, we have characterised the degree of bias potentially caused by misclassification in different parameter settings, finding that VE can be noticeably underestimated, particularly when the ratio between TD and ND cases in the study data is unbalanced. To address this potential bias, we developed multiple bias correction methods that provide unbiased VE estimates in both univariate and multivariate settings. When the test sensitivity and specificity are known or assumed, those values can be used to restore the true VE estimate by a relatively simple statistical procedure. Using simulations, we showed that our methods could successfully eliminate the bias in VE estimates obtained from misclassified data, although some uncertainty was introduced as a result of the information loss.

We believe that our methods could therefore enable researchers to report unbiased VE estimates even when imperfect tests had to be used. Such methods could also help in the scaling up of TND studies, as tests with limited performance are usually inexpensive and logistically convenient.

Although TND is a relatively new study design, first appearing in a publication in 2005 [27], it has gained broad popularity and is becoming a standard approach in VE studies. One of the largest factors that have contributed to its widespread use is the fact that data collection can be completed within clinical setups [1]. Whereas cohort or case–control studies usually require additional efforts including follow-up or recruitment of non-patients, TND studies only involve patients visiting healthcare facilities with suspects of certain diseases and thus routinely collected clinical data can be easily adapted for analysis. VE studies of influenza, for which TND is most frequently used, often use PCR as a diagnostic tool for better data quality [23]. However, such studies usually involve intensive effort and cost, and thus may only be feasible by large-scale research bodies. Our bias correction methods may open a possibility of wider use of clinical data especially in settings where rapid tests are routinely used for diagnosis. For example, rapid influenza diagnostic tests are routinely used for outpatient clinics and hospitals in Japan, and such clinical data have facilitated a number of TND studies [28–33]. Such studies based on rapid tests could benefit from our methods, as it would provide strong support for the validity of their estimates. Our methods may also be useful in resource-limited settings or for diseases without high-performance diagnostic tools.

Even in resourceful settings where high-performance tests are available, the slight possibility of misclassification might not always be neglected. Although PCR tests are currently used as a gold-standard for influenza diagnosis, their sensitivity and specificity may not be exact 100%; especially, the sensitivity of the test depends not only on microbiological technique but also on the quality of swab samples. In addition, it is suggested that the sensitivity of PCR tests may change during the time course of infection [34] and be sufficiently high only during a limited time window. Our simulation study also indicated that a high heterogeneity in individual characteristics in the study population might increase the bias. Our methods could enable researchers to implement sensitivity analysis by assuming the possible test sensitivity and specificity in such cases.

Our bias correction methods are also intended to be reasonably straightforward for researchers to introduce. Existing estimation tools including software libraries and packages are often used in epidemiological analyses. Incorporating the MO approach, our parametric bootstrapping bias correction method only involves data manipulation and does not require modification of the estimation algorithm. Once multiple sets of data are randomly generated, any type of analysis can be performed as long as the results can be summarised over the MO datasets. Of particular note is that our methods for multivariate analysis allow stratification of sensitivity and specificity among individuals. Therefore, the users can employ more complex misclassification mechanisms including time-varying test performance or test performance affected by individual characteristics. Datasets with a mixture of different diagnostic tools [3, 35] can also be handled by applying different values for each test.

There are some limitations to our study. We only focused on misclassification of diagnosis (i.e. misclassified outcomes) and did not consider misclassification of covariates (e.g. vaccine history and other confounding variables), which is another important type of misclassification in TND studies [10]. Further, it is generally not easy to plausibly estimate the sensitivity and specificity for measurement of covariates (e.g. recall bias), which must be known or assumed to implement bias correction. However, if reliable estimates are available, an extension of our approach may yield bias-corrected VE estimates in the presence of covariate misclassification. Moreover, to keep our focus only on diagnostic misclassification, our methods rested on the assumption that other sources of bias in TND studies are non-existent or properly addressed. Potential sources of bias in TND studies have been discussed elsewhere [16, 36, 37], and the researchers conducting TND studies need to carefully consider the possibility of such biases in addition to the diagnostic misclassification. Lastly, it must be noted that our methods depend on the assumed test sensitivity and specificity, and that misspecifying those values can result in an improper correction. The sensitivity and specificity of tests are usually reported by manufacturers in a comparison of the test results with gold-standard tests; however, when such gold-standard tests themselves are not fully reliable or when no available test has satisfactory performance to be regarded as gold-standard, specifying sensitivity and specificity of a test is in principle impossible. Further, test performances reported by manufacturers might lack sufficient sample size or might not be identical to those in the actual study settings. Use of composite reference standards [38, 39] or external/internal validation approaches [40] may help overcome these problems.

Although the presence of imperfect diagnosis limits the quality of clinical data, such data can still hold useful information, and this information can be transformed into useful insights by appropriate statistical processing. Our bias correction methods were developed primarily for TND studies, but a similar approach could be applied to broader classes of estimation problems with misclassification. Potential areas for future analysis include extension to test data involving continuous quantitative measurements, and coupling with dynamic transmission models. The value of routinely collected data in healthcare settings has become widely recognised with the advancement of data infrastructure, and we believe our methods could help support the effective use of such data.

## Conclusion

Bias correction methods for the TND studies were developed to address potential misclassification bias due to imperfect tests.

## Data Availability

This study did not involve original data collection and replication codes are available on GitHub under the MIT License (https://github.com/akira-endo/TND-biascorrection/).

## Footnotes

1 It has been suggested that a possible violation of this assumption occur as a result of virus interference [17], but conclusive evidence for this is currently lacking [18, 19] and the effect on VE estimates may be limited in any case [20].

2 This may not be true, for example, if vaccination reduces the severity of TD and hence reduces the likelihood of medical attendance.

3 For example, a case ratio of 0.5 indicates TD:ND = 1:1 in the unvaccinated. The value is smaller than 0.5 when TD < ND and greater than 0.5 when TD > ND.

4 Strictly speaking, proportion positive is a different quantity from case ratio, but it should serve as a reasonable proxy of the case ratio in most settings.

5 Young *et al*. [22] was not included because they did not report case counts.

## Supplementary material

For supplementary material accompanying this paper visit https://doi.org/10.1017/S0950268820002058. click here to view supplementary material

## Appendix

### Mathematical notations in section ‘Methods and results’

#### Characterising bias in TND studies

*r*_1_, *r*_0_: prevalence of TD/ND in the unvaccinated population
*n*_*V*_, *n*_*U*_: vaccinated and unvaccinated population size
*γ*: the relative risk of TD in the vaccinated population relative to the unvaccinated (VE = 1 − *γ*)
*m*_*V*_, *m*_*U*_: the probability of medical attendance given ND in vaccinated/unvaccinated population
*μ*: factor for the probability of medical attendance given TD in relative to that given ND
*x*_*V*_, *x*_*U*_, *x*_+*V*_, *x*_+*U*_, *x*_−*V*_, *x*_−*U*_, *y*_*V*_, *y*_*U*_, *y*_+*V*_, *y*_+*U*_, *y*_−*V*_, *y*_−*U*_: the expected number of cases in the population; see Table 1 for definitions
*X*_*V*_, *X*_*U*_, *Y*_*V*_, *Y*_*U*_: the observed case counts subject to misclassification
*q*: the proportion of study samples relative to the total population
*α*, *β*: sensitivity and specificity of the test
*C*: the classification matrix
*c*: the Youden index of the test; the determinant of *C δ*: odds of medically-attended TD in the unvaccinated population

#### Bias correction in univariate analysis

*γ**: corrected odds ratio
*σ**: shape parameter of the log-normal distribution that gives the confidence interval of *γ** (see Equation (4))
*λ*_*V*_, *λ*_*U*_: ‘baseline medical attendance’, the mean number of vaccinated/unvaccinated patients when vaccine has no effect (VE = 0)

#### Bias correction in multivariate analysis

*ξ* = (*ξ*^1^, *ξ*^2^, …, *ξ*^*n*^): covariates included in the model, where *xi*^1^ denotes vaccination history
*Z*_*i*_, *z*_*i*_: observed test result and true disease state for individual *i*
*p*_1_(*ξ*), *p*_0_(*ξ*): probability that the true test result is positive/negative for an individual with covariates *ξ*
*θ*, *θ**: model parameter for *p*_1_(*ξ*) and its estimate
probability for the observed test result *Z*_*i*_
imputed disease state of individual *i* in the *j*-th imputed dataset
estimated probability that the test result for individual *i* is incorrect
*ɛ*^*j*^: point estimate for VE from the *j*-th imputed dataset
