# Supplementary material for: Bias correction methods for test-negative designs in the presence of misclassification
Source: Epidemiol Infect. 2020 Sep 8;148:e216. doi: 10.1017/S0950268820002058 (PMC7522852; doi:10.1017/S0950268820002058)
Supplement: Supplementary file 1 [file S0950268820002058sup001.pdf]

SUPPLEMENTARY DOCUMENT: BIAS CORRECTION METHODS FOR TEST-NEGATIVE DESIGN IN THE PRESENCE OF MISCLASSIFICATION

Akira Endo, Sebastian Funk, Adam J. Kucharski

1 Expected degree of bias and parameter settings

Based on Equation (3) in the main text, we extensively explored the relationship between the bias in vaccine effectiveness (VE) estimates and the parameter settings. Figure S1 shows the expected degree of bias for different combinations of parameter values (sensitivity: α, specificity: β, true VE: $1-\gamma$ and case ratio: $\frac{\delta}{1+\delta}$). A nonlinear relationship can be seen in the contour plots. In general, the influence of sensitivity is larger in the high case ratio region, and that of specificity is larger in the low case ratio region. Both sensitivity and specificity affect the bias when the case ratio is intermediate. Interestingly, the true VE have different effects depending on the case ratio: high VE leads to larger bias when the case ratio is low, while moderate VE gives the largest bias when the case ratio is intermediate to high.

As the specificity of diagnostic tests tend to be higher than the sensitivity, we further explored the cases of higher specificity (> 95%) in Figure S2. Even if the specificity is sufficiently high, the level of bias can be larger than one might expect within the realistic range of case ratio (0.1-0.7).

2 Univariate model and parameter estimation

The sample size of a TND study is usually unconstrained as the study design requires every patient with TD-like symptoms to be included, where the study population is typically limited to patients visiting specific medical institutions and is sufficiently smaller than the total population. Therefore, it is natural that we assume that the reported incidence of TD and ND both follow Poisson-distributions. The mean number of unvaccinated patients in the dataset is given as $\lambda_U = qm_U(r_1\mu + r_0)n_U$. Let $\lambda_V = \frac{m_V n_V}{m_U n_U}\lambda_U$ so that λ_V corresponds to the mean number of vaccinated patients when $\gamma = 1$, i.e. VE = 0. This definition is to ensure that parameters γ and λ_V are mutually independent. Let $\delta = \frac{r_1\mu}{r_0}$ be the odds of the (medically-attended) target disease in the unvaccinated population. Using these four parameters $\gamma, \delta, \lambda_V, \lambda_U$, we get the following table for (potentially mis-classified) mean case counts:

	Vaccinated	Unvaccinated
Test positive	$\frac{\alpha\gamma\delta+(1-\beta)}{1+\delta}\lambda_V$	$\frac{\alpha\delta+(1-\beta)}{1+\delta}\lambda_U$
Test negative	$\frac{(1-\alpha)\gamma\delta+\beta}{1+\delta}\lambda_V$	$\frac{(1-\alpha)\delta+\beta}{1+\delta}\lambda_U$
Subtotal	$\frac{1+\gamma\delta}{1+\delta}\lambda_V$	λ_U

When data $D = (X_V, Y_V, X_U, Y_U)$ is obtained following this misclassified pattern, we can construct the likelihood of obtaining such data, given underlying parameters, as

$$\begin{aligned}\mathcal{L}(\gamma,\delta,\lambda_V,\lambda_U;D) = \text{Pois}\left(X_V;\frac{\alpha\gamma\delta+(1-\beta)}{1+\delta}\lambda_V\right)\text{Pois}\left(Y_V;\frac{(1-\alpha)\gamma\delta+\beta}{1+\delta}\lambda_V\right)\\ \text{Pois}\left(X_U;\frac{\alpha\delta+(1-\beta)}{1+\delta}\lambda_U\right)\text{Pois}\left(Y_U;\frac{(1-\alpha)\delta+\beta}{1+\delta}\lambda_U\right).\end{aligned} \quad \text{(S1)}$$

By expanding this equation we get

$$\begin{aligned}&\mathcal{L}(\gamma,\delta,\lambda_V,\lambda_U;D)\\&=\frac{[\alpha\gamma\delta+(1-\beta)]^{X_V}[(1-\alpha)\gamma\delta+\beta]^{Y_V}[\alpha\delta+(1-\beta)]^{X_U}[(1-\alpha)\delta+\beta]^{Y_U}\lambda_V^{S_V}\lambda_U^{S_U}}{(1+\delta)^{S_V}(1+\delta)^{S_U}X_V!Y_V!X_U!Y_U!\exp\left(\frac{1+\gamma\delta}{1+\delta}\lambda_V\right)\exp\left(\lambda_U\right)},\end{aligned}\tag{S2}$$

where $S_V = X_V + Y_V$ and $S_U = X_U + Y_U$.

For mathematical convenience, we change the variable λ_V to $\lambda_V^{'} = \frac{1+\gamma\delta}{1+\delta}\lambda_V$. Let $l = \log\mathcal{L}(\gamma,\delta,\lambda_V^{'},\lambda_U;X)$. Partial derivatives of l are

$$\begin{aligned}\frac{\partial l}{\partial\gamma}&=\frac{\alpha\delta X_V}{\alpha\gamma\delta+(1-\beta)}+\frac{(1-\alpha)\delta Y_V}{(1-\alpha)\gamma\delta+\beta}-\frac{\delta S_V}{1+\gamma\delta}\\\frac{\partial l}{\partial\delta}&=\frac{\alpha\gamma X_V}{\alpha\gamma\delta+(1-\beta)}+\frac{(1-\alpha)\gamma Y_V}{(1-\alpha)\gamma\delta+\beta}+\frac{\alpha X_U}{\alpha\delta+(1-\beta)}+\frac{(1-\alpha)Y_U}{(1-\alpha)\delta+\beta}-\frac{\gamma S_V}{1+\gamma\delta}-\frac{S_U}{1+\delta}\\\frac{\partial l}{\partial\lambda_V^{'}}&=\frac{S_V}{\lambda_V^{'}}-1\\\frac{\partial l}{\partial\lambda_U}&=\frac{S_U}{\lambda_U}-1\end{aligned}\tag{S3}$$

Equation (S3) gives the maximum likelihood estimates:

$$\begin{aligned}\gamma^*&=\frac{X_V-\frac{1-\beta}{\beta}Y_V}{Y_V-\frac{1-\alpha}{\alpha}X_V}\cdot\frac{Y_U-\frac{1-\alpha}{\alpha}X_U}{X_U-\frac{1-\beta}{\beta}Y_U}\\\delta^*&=\frac{\beta}{\alpha}\cdot\frac{X_U-\frac{1-\beta}{\beta}Y_U}{Y_U-\frac{1-\alpha}{\alpha}X_U}\\\lambda_V^{'*}&=S_V\\\lambda_U^*&=S_U\end{aligned}\tag{S4}$$

The confidence intervals for parameters can be constructed using the Fisher's information matrix from Equation (S3). $\lambda_V^{'}$ and λ_U are independent from other parameters and

$$\mathrm{Var}(\lambda_V^{'})=-\frac{\partial^2 l}{\partial\lambda_V^{'2}}=\frac{S_V}{\lambda_V^{'2}}-\frac{\partial^2 l}{\partial\lambda_U^2}=\frac{S_U}{\lambda_U^2}\tag{S5}$$

We log-transform γ and δ for mathematical convenience. Noting that $\frac{\partial v}{\partial(\log u)} = u\frac{\partial v}{\partial u}$, we get

$$\begin{aligned}-\frac{\partial^2 l}{\partial(\log(\gamma))^2}=&\frac{\gamma\delta}{(1+\gamma\delta)^2}S_V-\frac{\alpha(1-\beta)\gamma\delta}{[\alpha\gamma\delta+(1-\beta)]^2}X_V-\frac{(1-\alpha)\beta\gamma\delta}{[(1-\alpha)\gamma\delta+\beta]^2}Y_V\\-\frac{\partial^2 l}{\partial\log\gamma\partial\log\delta}=&\frac{\gamma\delta}{(1+\gamma\delta)^2}S_V-\frac{\alpha(1-\beta)\gamma\delta}{[\alpha\gamma\delta+(1-\beta)]^2}X_V-\frac{(1-\alpha)\beta\gamma\delta}{[(1-\alpha)\gamma\delta+\beta]^2}Y_V\\-\frac{\partial^2 l}{\partial(\log\delta)^2}=&\frac{\gamma\delta}{(1+\gamma\delta)^2}S_V-\frac{\alpha(1-\beta)\gamma\delta}{[\alpha\gamma\delta+(1-\beta)]^2}X_V-\frac{(1-\alpha)\beta\gamma\delta}{[(1-\alpha)\gamma\delta+\beta]^2}Y_V\\&+\frac{\delta}{(1+\delta)^2}S_U-\frac{\alpha(1-\beta)\delta}{[\alpha\delta+(1-\beta)]^2}X_U-\frac{(1-\alpha)\beta\delta}{[(1-\alpha)\delta+\beta]^2}Y_U\end{aligned}\tag{S6}$$

With the parameter values estimated in Eq. (S4), we get the following information matrix

$$\begin{bmatrix}\frac{\hat{x}_V\hat{y}_V}{S_V}\left[1-S_V\left(\frac{\alpha(1-\beta)}{X_V}+\frac{(1-\alpha)\beta}{Y_V}\right)\right] & \frac{\hat{x}_V\hat{y}_V}{S_V}\left[1-S_V\left(\frac{\alpha(1-\beta)}{X_V}+\frac{(1-\alpha)\beta}{Y_V}\right)\right]\\\frac{\hat{x}_V\hat{y}_V}{S_V}\left[1-S_V\left(\frac{\alpha(1-\beta)}{X_V}+\frac{(1-\alpha)\beta}{Y_V}\right)\right] & \frac{\hat{x}_V\hat{y}_V}{S_V}\left[1-S_V\left(\frac{\alpha(1-\beta)}{X_V}+\frac{(1-\alpha)\beta}{Y_V}\right)\right]+\frac{\hat{x}_U\hat{y}_U}{S_U}\left[1-S_U\left(\frac{\alpha(1-\beta)}{X_U}+\frac{(1-\alpha)\beta}{Y_U}\right)\right]\end{bmatrix}$$

where $\hat{x}_\xi = \frac{1}{c}[\beta X_\xi - (1-\beta)Y_\xi]$ and $\hat{y}_\xi = \frac{1}{c}[\alpha Y_\xi - (1-\alpha)X_\xi]$ are the estimated true case counts for $\xi = V, U$. Let $p_V = x_V/(x_V + y_V)$ and $p_U = x_U/(x_U + y_U)$ be the corresponding true binomial probabilities.

The inverse of the information matrix provides variance of estimates: in particular, for $\log\gamma$ we get

$$\begin{aligned}\mathrm{Var}(\log\gamma^*) &= \frac{S_V}{x_V y_V}\cdot\frac{1}{\left[1-\left(\frac{\alpha(1-\beta)}{\pi_V}+\frac{(1-\alpha)\beta}{1-\pi_V}\right)\right]}+\frac{S_U}{x_U y_U}\cdot\frac{1}{\left[1-\left(\frac{\alpha(1-\beta)}{\pi_U}+\frac{(1-\alpha)\beta}{1-\pi_U}\right)\right]}\\ &= \frac{S_V}{x_V y_V}\cdot\frac{\pi_V(1-\pi_V)}{(1-\pi_V-(1-\alpha))(\pi_V-(1-\beta))}+\frac{S_U}{x_U y_U}\cdot\frac{\pi_U(1-\pi_U)}{(1-\pi_U-(1-\alpha)(\pi_U-(1-\beta))}\\ &= \frac{c^2}{S_V}\frac{\pi_V(1-\pi_V)}{(1-\pi_V-(1-\alpha))^2(\pi_V-(1-\beta))^2}+\frac{c^2}{S_U}\frac{\pi_U(1-\pi_U)}{(1-\pi_U-(1-\alpha))^2(\pi_U-(1-\beta))^2},\end{aligned} \tag{S7}$$

equivalent to the Eq. (7) in the main text. We can relate this to the true standard error that would be obtained with perfect tests,

$$\mathrm{SD}(\log\gamma_{\text{true}}) = \sqrt{\frac{1}{S_V p_V(1-p_V)}+\frac{1}{S_U p_U(1-p_U)}} = \sqrt{\frac{{\sigma_V}^2}{S_V}+\frac{{\sigma_U}^2}{S_U}}, \tag{S8}$$

or to the observed standard error (without correction),

$$\mathrm{SD}(\log\gamma_{\text{raw}}) = \sqrt{\frac{1}{S_V \pi_V(1-\pi_V)}+\frac{1}{S_U \pi_U(1-\pi_U)}} = \sqrt{\frac{{\Sigma_V}^2}{S_V}+\frac{{\Sigma_U}^2}{S_U}}, \tag{S9}$$

where $\sigma_V = [p_V(1-p_V)]^{-1/2}$ and $\sigma_U = [p_U(1-p_U)]^{-1/2}$ are the components of the true standard error and $\Sigma_V = [\pi_V(1-\pi_V)]^{-1/2}$ and $\Sigma_U = [\pi_U(1-\pi_U)]^{-1/2}$ are those of uncorrected standard error. We get

$$\begin{aligned}\sigma^* = \mathrm{SD}(\log(\gamma^*)) &= \sqrt{\frac{\sigma_V^2}{S_V}\cdot\frac{1}{\left(1-\frac{1-\alpha}{1-\pi_V}\right)\left(1-\frac{1-\beta}{\pi_V}\right)}+\frac{\sigma_U^2}{S_U}\cdot\frac{1}{\left(1-\frac{1-\alpha}{1-\pi_U}\right)\left(1-\frac{1-\beta}{\pi_U}\right)}}\\ &= \frac{1}{c}\sqrt{\frac{\Sigma_V^2}{S_V}\cdot\left(\frac{\pi_V(1-\pi_V)}{p_V(1-p_V)}\right)^2+\frac{\Sigma_U^2}{S_U}\cdot\left(\frac{\pi_U(1-\pi_U)}{p_U(1-p_U)}\right)^2}.\end{aligned} \tag{S10}$$

This equation indicates that the confidence intervals diverge when the true outcome is bipolarised ($p_V, p_U \simeq 0$ or 1).

3 Multivariate model and likelihood

Suppose that covariates $\xi = (\xi^1, \xi^2, ..., \xi^n)$ are included in the model, and that ξ^1 corresponds to vaccination history (1: vaccinated, 0: unvaccinated). ξ is expected to have a certain distribution over the total population N, and let us denote the frequency density of covariates ξ by $N(\xi)$, where $\int N(\xi)d\xi = N$. Let $\rho_1(\xi)$ and $\rho_0(\xi)$ be the conditional probabilities that an individual is included in the study with TD and ND, respectively, given covariates ξ. Incorporating misclassification, the probability of an individual i with covariates ξ_i being included and tested positive/negative will be

$$\begin{aligned}\rho_+(\xi_i) &= \alpha\rho_1(\xi_i) + (1-\beta)\rho_0(\xi_i)\\ \rho_-(\xi_i) &= (1-\alpha)\rho_1(\xi_i) + \beta\rho_0(\xi_i)\end{aligned} \tag{S11}$$

Assuming that disease incidences follow Poisson distributions, as in the univariate case, we can obtain the probability density of observing data $D = \{Z_i, \xi_i\}_{i=1,2,...S}$ (Z_i denotes the test result of individual i) as

$$\mathcal{P}(D) = \mathrm{Pois}(S_+;\lambda_+)\,\mathrm{Pois}(S_-;\lambda_-)\prod_{i\in\{+\}}\frac{\rho_+(\xi_i)N(\xi_i)}{\lambda_+}\prod_{i\in\{-\}}\frac{\rho_-(\xi_i)N(\xi_i)}{\lambda_-}. \tag{S12}$$

where λ_+ and λ_- are the mean incidence of being included in the study and tested positive/negative: $\lambda_\pm = \int \rho_\pm(\xi) N(\xi) d\xi$. The first two Poisson distributions on the right-hand side of Eq. (S12) give the probability that the study yields S_+ positive and S_- negative subjects. The products that follow represent the probability density for covariates ξ_i observed in the positive/negative group.

Suppose that we model this system using a parameter set θ. We could directly model $\rho_1(\xi_i;\theta)$ and $\rho_0(\xi_i;\theta)$; however, it is often more convenient to model the binomial probability for the true outcome $p_1(\xi_i) = \frac{\rho_1(\xi_i)}{\rho(\xi_i)}$ and $p_0(\xi_i) = \frac{\rho_0(\xi_i)}{\rho(\xi_i)}$, where $\rho(\xi_i) = \rho_1(\xi_i) + \rho_0(\xi_i) = \rho_+(\xi_i) + \rho_-(\xi_i)$ is the probability density of being included in the study given covariates ξ, because the absolute scale of incidence is rarely of a primary concern. The binomial probabilities for the respective observed outcomes (with errors) are then given by:

$$\begin{aligned} \pi_+(\xi_i;\theta) &= \alpha p_1(\xi_i;\theta) + (1-\beta) p_0(\xi_i;\theta) \\ \pi_-(\xi_i;\theta) &= (1-\alpha) p_1(\xi_i;\theta) + \beta p_0(\xi_i;\theta) \end{aligned} \tag{S13}$$

Let us use parameter set θ to model the binomial probabilities π_+ (and π_-) and assume that another set of parameters η (nuisance parameters) characterise $\rho(\xi_i)$. Then our objective is reduced to the estimation of θ and η.

Rearranging Equation (S12), we get the joint likelihood for θ and η:

$$\mathcal{L}(\theta,\eta;D) = \binom{S}{S_+} \mathrm{Pois}(S;\lambda(\eta)) \prod_{i=1}^{S} \frac{\rho(\xi_i;\eta) N(\xi_i)}{\lambda(\eta)} \prod_{i=1}^{S} \pi_{Z_i}(\xi_i;\theta), \tag{S14}$$

where $\lambda(\eta)$ is the overall mean incidence: $\lambda(\eta) = \int \rho(\xi;\eta) N(\xi) d\xi$. The factor outside the products on the right-hand side of Eq. (S14) is the probability that the study yields S subjects of which S_+ are positives and S_- are negatives. The first product is the probability density for covariates ξ_i observed in data D, and the second product is the binomial probabilities for the test results Z_i. When only θ is of our concern, we can obtain the MLE for θ by maximising

$$\mathcal{L}(\theta;D) = \prod_{i=1}^{S} \pi_{Z_i}(\xi_i;\theta) = \prod_{i\in\{+\}} [\alpha p_1(\xi_i;\theta) + (1-\beta) p_0(\xi_i;\theta)] \prod_{i\in\{-\}} [(1-\alpha) p_1(\xi_i;\theta) + \beta p_0(\xi_i;\theta)], \tag{S15}$$

as θ and η are separate in the likelihood (S14).

4 Increased uncertainty introduced by misclassification

Although our bias correction methods provide unbiased VE estimates from potentially misclassified test results, the resulting uncertainty is larger than that which would be obtained from estimates using the true disease status. In Figure S3, we compared bias-corrected estimates obtained from misclassified data (by the direct likelihood method in the multivariate setting) with those obtained from the true data (i.e., 100% sensitivity and specificity). Although both estimates are unbiased around the true value, the results from the misclassified data exhibit higher variability (by a factor of 1.1-3.0) due to the loss of information caused by misdiagnosis. Increased uncertainty due to misclassification should be carefully considered when one calculates the power of test-negative design studies. Overestimated test performance may not only underestimate the true VE but also lead to overconfidence.

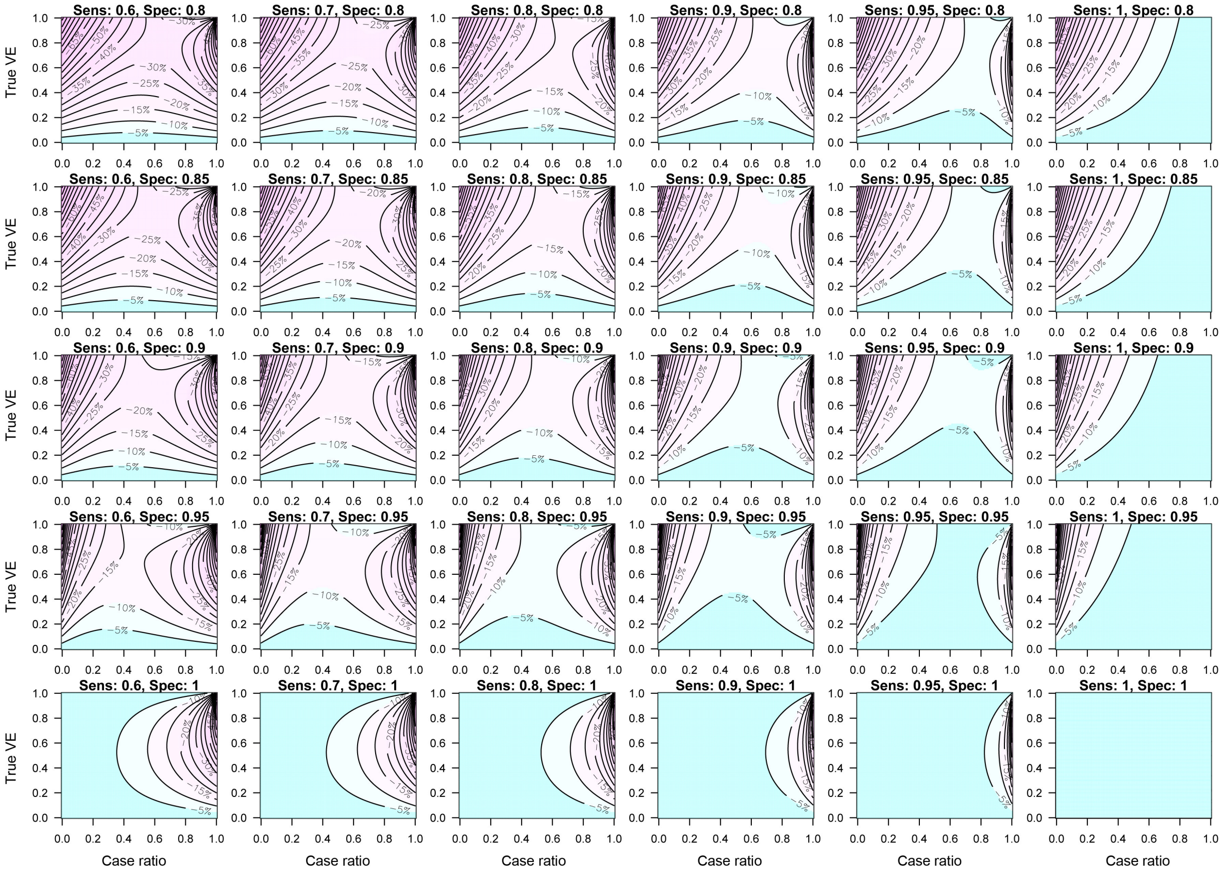

Figure S1: Bias in VE estimates caused by misclassification. Absolute bias (difference between the estimated VE and the true VE) for a set of parameter values is displayed in contour plots in percentage points. Negative figures indicate that the estimated VE is lower than the true VE.

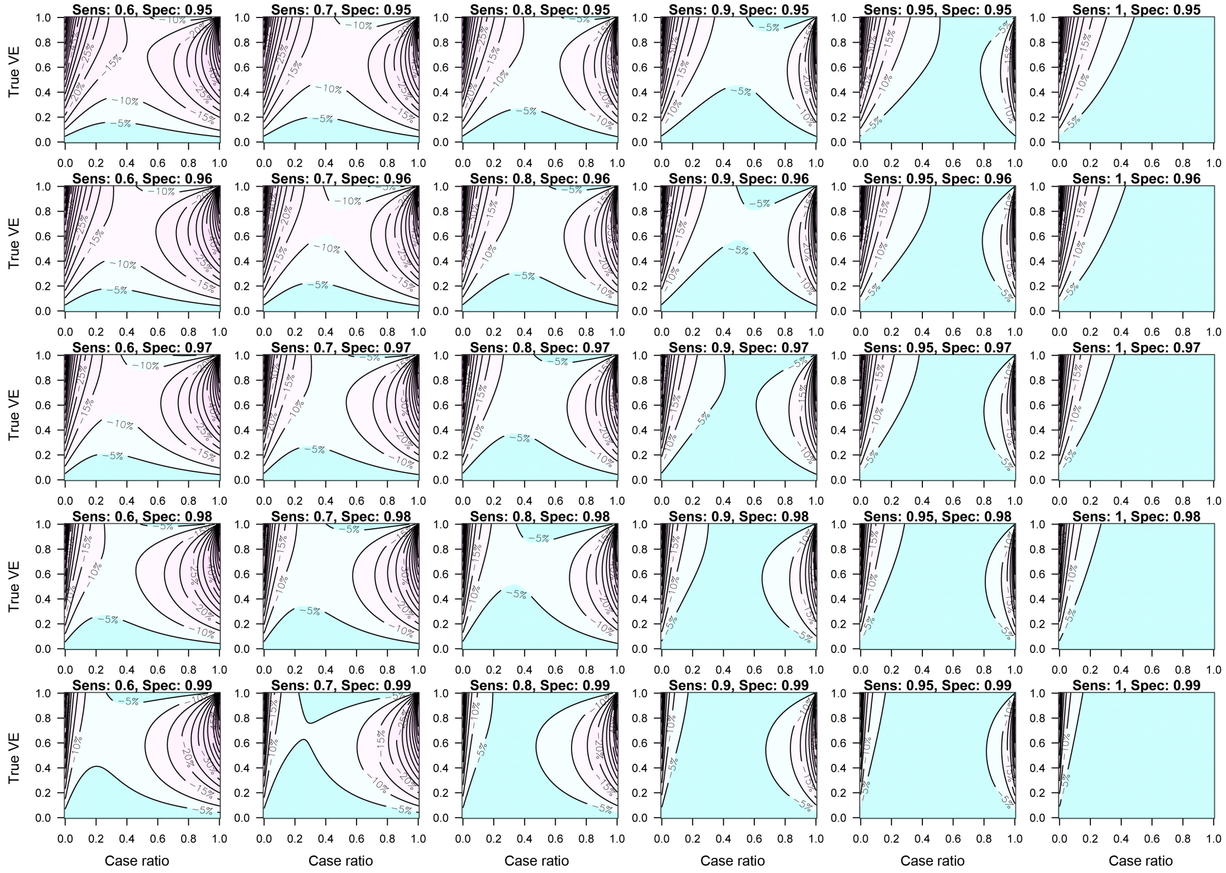

Figure S2: Bias in VE estimates caused by misclassification (high specificity). Absolute bias (difference between the estimated VE and the true VE) for a set of parameter values is displayed in contour plots in percentage points. Negative figures indicate that the estimated VE is lower than the true VE.

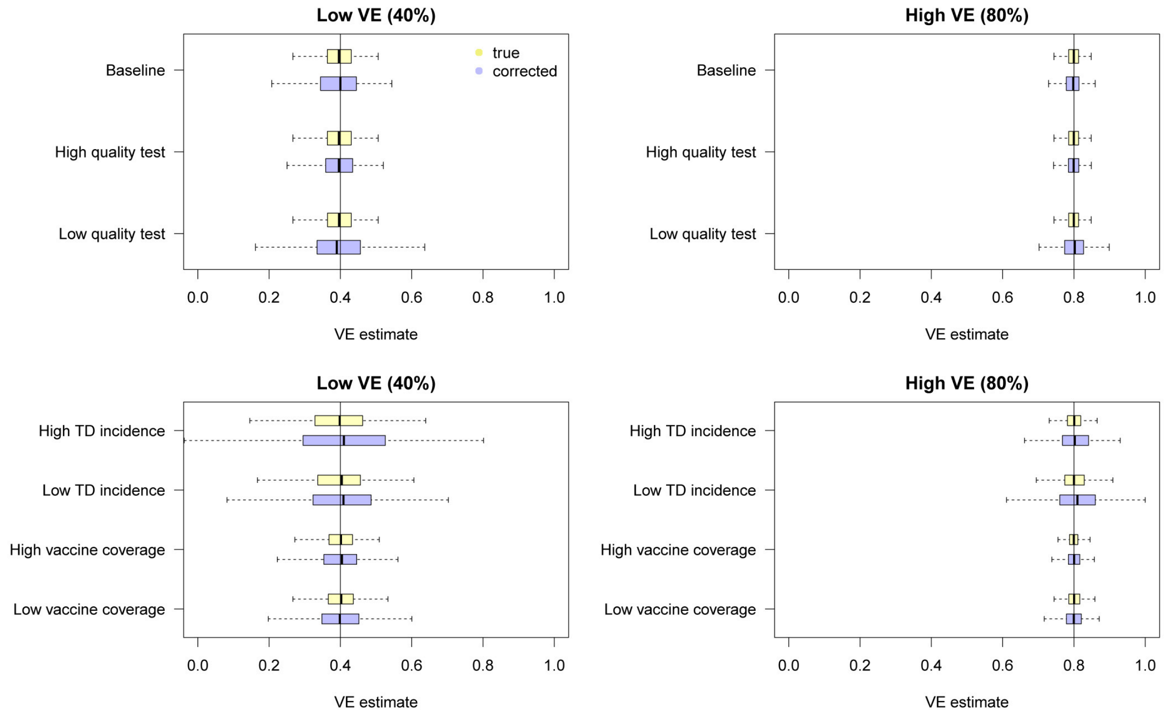

Figure S3: Uncertainty in VE estimates obtained from the true/misclassified datasets in the multivariate setting. The distributions of VE estimates from the simulated true (yellow) and misclassified (light blue) data are shown. The direct likelihood method was employed to correct biases in the misclassified data.
